# Large-scale epidemiological analysis of common skin diseases to identify shared and unique comorbidities and demographic factors

**DOI:** 10.3389/fimmu.2023.1309549

**Published:** 2024-01-08

**Authors:** Qinmengge Li, Matthew T. Patrick, Sutharzan Sreeskandarajan, Jian Kang, J. Michelle Kahlenberg, Johann E. Gudjonsson, Zhi He, Lam C. Tsoi

**Affiliations:** ^1^ Department of Biostatistics, University of Michigan, Ann Arbor, MI, United States; ^2^ Department of Dermatology, University of Michigan, Ann Arbor, MI, United States; ^3^ The Center for Autoimmune Genomics and Etiology, Cincinnati Children’s Hospital Medical Center, Cincinnati, OH, United States; ^4^ Rheumatology, Internal Medicine, University of Michigan, Ann Arbor, MI, United States; ^5^ Department of Computational Medicine and Bioinformatics, University of Michigan, Ann Arbor, MI, United States

**Keywords:** epidemiology, claims, skin disease, comorbidity, Optum

## Abstract

**Introduction:**

The utilization of large-scale claims databases has greatly improved the management, accessibility, and integration of extensive medical data. However, its potential for systematically identifying comorbidities in the context of skin diseases remains unexplored.

**Methods:**

This study aims to assess the capability of a comprehensive claims database in identifying comorbidities linked to 14 specific skin and skin-related conditions and examining temporal changes in their association patterns. This study employed a retrospective case-control cohort design utilizing 13 million skin/skin-related patients and 2 million randomly sampled controls from Optum’s de-identified Clinformatics^®^ Data Mart Database spanning the period from 2001 to 2018. A broad spectrum of comorbidities encompassing cancer, diabetes, respiratory, mental, immunity, gastrointestinal, and cardiovascular conditions were examined for each of the 14 skin and skin-related disorders in the study.

**Results:**

Using the established type-2 diabetes (T2D) and psoriasis comorbidity as example, we demonstrated the association is significant (P-values<1x10^-15^) and stable across years (OR=1.15-1.31). Analysis of the 2014-2018 data reveals that celiac disease, Crohn’s disease, and ulcerative colitis exhibit the strongest associations with the 14 skin/skin-related conditions. Systemic lupus erythematosus (SLE), leprosy, and hidradenitis suppurativa show the strongest associations with 30 different comorbidities. Particularly notable associations include Crohn’s disease with leprosy (odds ratio [OR]=6.60, 95% confidence interval [CI]: 3.09-14.08), primary biliary cirrhosis with SLE (OR=6.07, 95% CI: 4.93-7.46), and celiac disease with SLE (OR=6.06, 95% CI: 5.49-6.69). In addition, changes in associations were observed over time. For instance, the association between atopic dermatitis and lung cancer demonstrates a marked decrease over the past decade, with the odds ratio decreasing from 1.75 (95% CI: 1.47-2.07) to 1.02 (95% CI: 0.97-1.07). The identification of skin-associated comorbidities contributes to individualized healthcare and improved clinical management, while also enhancing our understanding of shared pathophysiology. Moreover, tracking these associations over time aids in evaluating the progression of clinical diagnosis and treatment.

**Discussion:**

The findings highlight the potential of utilizing comprehensive claims databases in advancing research and improving patient care in dermatology.

## 1 Introduction

Dermatological disorders are among the most common human diseases: more than a third of the global population suffers from some form of skin condition (1–5). While most skin disorders are not fatal, the burden on patients and society is severe; in fact, skin disorders are ranked the fourth leading cause of nonfatal disease burden globally (1). For instance, in a previous study, 60% of working patients noted significant work time lost, and 40% of non-working patients attributed their lack of work to psoriasis (6). In 1984, it was estimated that the cost for 2.3 million psoriasis outpatients in the US reached $1.5 billion per year (7), and a recent study reviewing the yearly cost for psoriasis nationwide increased the estimate to a range between $51.7 and $63.2 billion (8). Atopic dermatitis (AD) is another common skin condition that affects over 30 million patients in the US with a total annual cost of $4.2 billion in 2004 and $5.4 billion in 2016 (9). Although systemic lupus erythematosus (SLE), in which up to 70% patients exhibit skin manifestations, is relatively less common with a prevalence rate of around 10 per 10,000 in the US (10), the economic burden is significant, with a total annual cost estimated to be $13,735-$20,926 per patient (11). With these significant medical burden for the wide spectrum of dermatological disorders (12), the prevention and treatment of these conditions are critical issues for public health.

The associated comorbidities (i.e. co-occurrence of two different diseases (13)) for skin conditions contribute significantly to health and social burden. Numerous studies have found that skin disorders can be early manifestations of systemic diseases (13). Thus, it is important to assess patients’ risk for having other conditions in addition to their primary skin disorder; furthermore, understanding skin-associated comorbidities can further the development of better healthcare management (14) by facilitating early diagnosis of associated systemic conditions (13). Comorbidity information can also advance the identification of shared pathophysiology and risk factors, which play an important role in preventive medicine. For instance, cardiovascular disease has been found to have a significant association with psoriasis and contributes largely to the 5-year shorter life expectancy of psoriatic patients (15). Although this connection has been well publicized, a survey conducted between 2009 to 2012 showed that many physicians were unaware of this association potentially increasing the risk of delayed diagnosis and inadequate treatment of the associated cardiovascular comorbidity (16, 17).

While small cohort studies have been conducted to identify associated demographic variables or co-occurring conditions for specific skin-diseases (4, 18, 19) and the availability of large-scale claims databases has advanced precision medicine and comorbidity identification (20), limited research has investigated the potential of using these resources to identify, in a systematic fashion, associated skin conditions and comorbidities. A prominent claims data system is Optum’s de-identified Clinformatics^®^ Data Mart Database (CDM) (21, 22), an organized medical claims database that supports large-scale retrospective cohort studies. By utilizing medical records dating from 2001 to 2018, we revealed specific/shared comorbidities for 14 different skin diseases. With the 18-year time span, the trajectory of disease-comorbidity associations was also studied (23).

Our work highlights that most of the potential skin/skin-related condition-comorbidity pairs are positively associated. We calculated the trend of the skin-comorbidity associations over time and illustrated that the association between type-2 diabetes (T2D) and psoriasis over time is significant, stable, and consistent with previously published studies, confirming the validity of using CDM data in the identification of skin/skin-related disease comorbidities. However, analysis of some disease conditions can be biased, for instance, the association between psoriatic arthritis (PsA) and rheumatoid arthritis (RA) can be inflated when using unrestricted CDM data. This observation manifests potential misdiagnosis for some disease pairs in claims data. The CDM data processing and analyses in skin disease comorbidity identification can help inform the potentials and challenges in using large-scale claims data to study comorbidities and facilitate the development of individualized health care and optimization of clinical management.

## 2 Materials and methods

### 2.1 Data preparation

The data used in this study comes from CDM (21), a de-identified patient-level database provided by Optum, a national healthcare management company. The CDM database includes medical claims from various sources, including commercially insured patients, administrative services only patients, legacy medicare choice patients prior to 2006, and medicare advantage patients after 2006. It covers a span of 18 years, from 2001 to 2018, and includes over 63 million patients from all 50 U.S. states. However, the CDM cohort does not include patients insured by Medicaid, so the socioeconomic spectrum of the entire U.S. population is not fully represented in this dataset (22).

**Figure 1:**
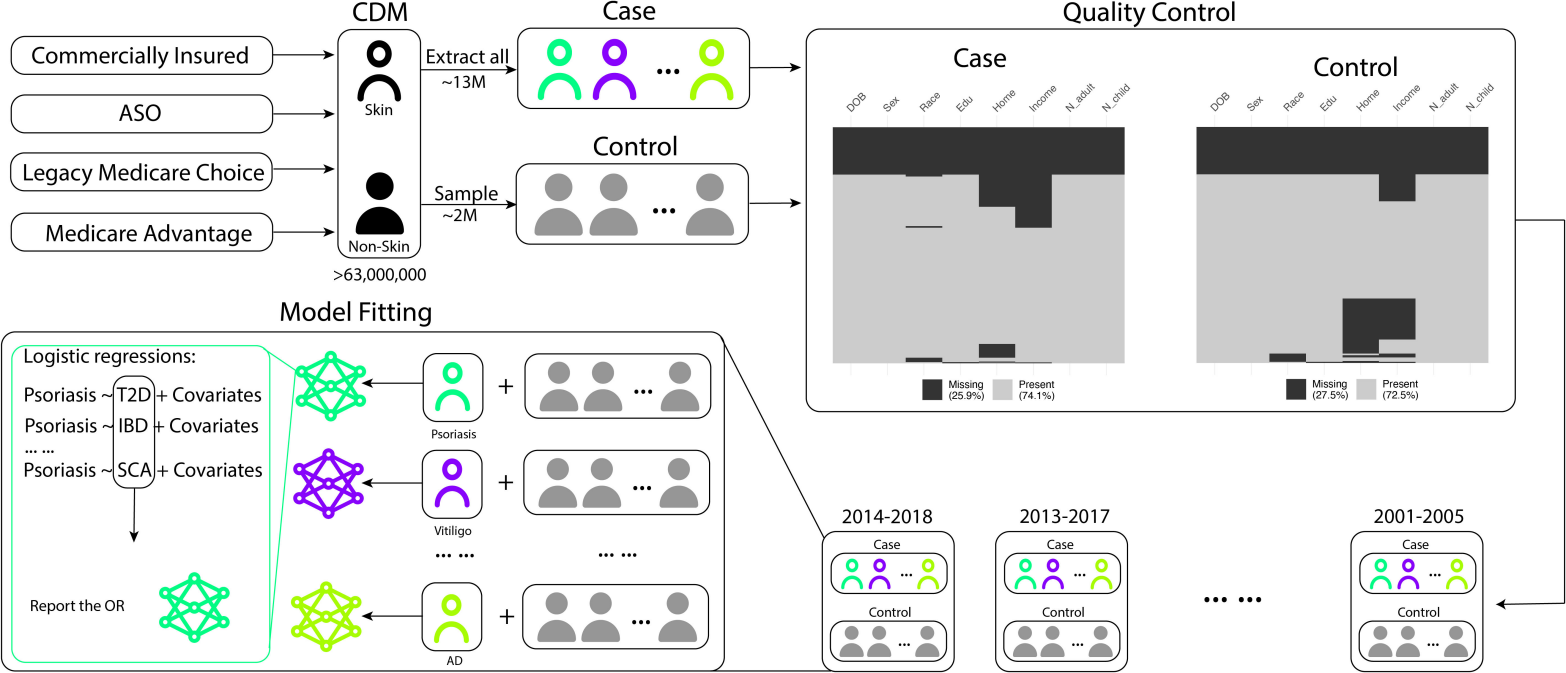
Data preprocessing and model fitting workflow. The flowchart illustrates the selection process for patients with skin-related conditions and the control group. Patients with the 14 skin-related conditions are initially extracted, and a separate control group of 2,000,000 patients is randomly sampled from the remaining cohort. Quality control steps are applied to remove patients with incomplete records. The subsequent statistical analyses involve comparing the extracted skin condition patients with the randomly sampled non-skin condition patients as the control group (psoriasis is used as an example in the above pipeline).

Our analysis focused on identifying comorbidities related to skin diseases. We began by selecting a total of 13,934,335 patients with at least one of the 14 skin conditions. These conditions were categorized into three groups: immune-mediated skin diseases (acne, rosacea, alopecia areata, vitiligo, psoriasis, atopic dermatitis, hidradenitis suppurativa, prurigo nodularis), non-immune-mediated skin diseases (aging, leprosy, pigmentation, melanoma), and skin-related disorders (systemic lupus erythematosus, psoriatic arthritis). For the control group, we randomly sampled 2 million unique patients from the entire CDM database, excluding those with any of the aforementioned 14 skin/skin-related diseases. We extracted and adjusted several demographic and socioeconomic variables for analysis, including age, sex, race, education level, income level, home ownership, and the number of adults and children in the household, to account for the higher socioeconomic sampling bias. To account for non-recorded comorbidities resulting from patients leaving the healthcare system, we also included the length of time patients stayed in the system as a covariate. In the subsequent analysis, we only included individuals with complete demographic and socioeconomic information leaving 7,553,273 patients and 726,230 controls. If a patient was diagnosed with two diseases within a 5-year time span, we considered those conditions to be co-occurring. This time range is based on empirical observations of the duration patients stay in the CDM system. We divided the full dataset into consecutive 5-year subsets (e.g., 2001-2005, 2002-2006,…, 2014-2018) and conducted separate analyses for each time interval. **Figure 1** provides an overview of our study.

### 2.2 Statistical analysis

A descriptive analysis was performed to provide an overview of the dataset and the distribution of all covariates. Categorical variables such as sex, race, education level, home ownership, and income level were summarized as percentages for each category. Continuous/Integer variables such as age, the number of children, and the number of adults in the household were summarized as mean values with their corresponding standard deviations.

Logistic regression was employed to model the association between each skin disease and comorbidity pair while accounting for potential confounding covariates. Treating either skin/skin-related disorders or comorbidities as outcome variable can achieve this goal. Since the other aim of this work is to model the risk of skin/skin-related disorders, therefore, in the following analysis we treat skin/skin-related disorders as outcome variable and comorbidities and other demographics as predictors. Age was categorized into specific ranges (e.g., <10, 10-20, 20-30,…, 70-80, >80), allowing for non-linear patterns, with the reference category being age<10. As weight and height information was unavailable, the obesity diagnosis code was used as a surrogate to control for the impact of low or high BMI on disease associations. Male and European ancestry were chosen as the reference categories for sex and race, respectively. Education level was categorized as “below high school,” “high school,” “bachelor,” and “above bachelor,” with “below high school” as the reference category. Annual household income was categorized as “<$40k,” “$40k-$49k,” “$50k-$59k,” “$60k-$74k,” “$75k-$99k,” and “$100k>,” with “<$40k” as the reference category. The time lengths for each patient in the system were calculated as the number of years between the first and last recorded diagnosis. For patient *i*, the logistic regression model for the following comorbidity analysis is thus:


$$\begin{array}{c} logit\left\{\mathrm{Pr}\left(Skin_{i}|X_{i}\right)\right\}\\ \;=\beta_{0}+\beta_{comorbidity}\times X_{i\mathrm{comorbidity}}+\beta_{obesity}\times X_{\mathrm{iobesity}}+\beta_{age}\\ \;\times X_{\mathrm{iage}}+\beta_{sex}\times X_{\mathrm{isex}}+\beta_{race}\times X_{\mathrm{irace}}+\beta_{education}\times X_{\mathrm{ieducation}}\\ \;+\beta_{income}\times X_{\mathrm{iincome}}+\beta_{child}\times X_{\mathrm{ichild}}+\beta_{adult}\times X_{\mathrm{iadult}}+\beta_{time}\times X_{\mathrm{itime},} \end{array}$$

where $\beta_{comorbidity}$ is the parameter of interest indicating the association levels for a pair of skin/skin-related condition and comorbidity, which can be interpreted as the log odds ratio of developing the skin/skin-related disease between patients with or without the comorbidity.

## 3 Results

### 3.1 Summary statistics

The summary information for the cases and controls during the period of 2014-2018 is presented in **Table 1** in addition to the US general population characteristics. When comparing the randomly controlled samples with the US general population, the CDM data represents older, higher income and education US population with less ethnic minorities. This further justifies controlling the socioeconomic factors in the logistic regression model for subsequent analysis. Consistent with previous studies (24–28), certain skin or skin-related disorders show a higher prevalence among women. For example, rosacea, alopecia areata, SLE, acne, and hidradenitis suppurativa (HS) have 67.6%, 73.7%, 86.3%, 67.6%, and 72.5% female patients, respectively, compared to 50.7% in the control group. We also found a higher proportion of European ancestry associated with the diagnosis of rosacea, aging (chronic exposure to sun or non-ionizing radiation), melanoma, and pigmentation (e.g. hyperpigmentation and freckles; detailed definition can be found in **Supplementary Table 1**), with percentages of 82.6%, 87.6%, 88.7%, and 81.8%, respectively, compared to the baseline composition of 72.2% Europeans in the control population. Conversely, the Hispanic and African American populations have lower proportions in most skin diseases compared to the control group, except for vitiligo (16.5%) and leprosy (14.5%) among Hispanics (control: 12.5%), and SLE (15.5%) and HS (18.6%) among African Americans (control: 10.5%). Patients of Asian heritage have a lower proportion of melanoma (0.9%) but a higher proportion of vitiligo (7.4%) and leprosy (8.9%) compared to the control group (4.8%). Furthermore, we observed that a higher education level is associated with a larger number of medical claims for skin disorders. Rosacea (30.5% above college), acne (35.4% above college), and pigmentation (30.1% above college) have the most significant elevation compared to the control group (18.8% above college). Similarly, a higher income level is linked to a stronger association with medical claims for skin conditions, with rosacea (53.4% income >$100k), acne (61.0% >$100k), and pigmentation (53.1% >$100k) showing the largest contrast compared to the control population (39.2% >$100k).

**Table 1:** Descriptive analysis for CDM data.

		PN	Rosacea	AD	PsA	Psoriasis	Alopecia areata	Vitiligo	SLE	Acne	Aging	Melanoma	Pigmentation	Leprosy	HS	Control	Overall Skin	US population
N		146,796	351,026	1,458,417	48,241	272,913	108,462	31,914	67,718	801,150	480,415	73,928	1,297,949	235	36,364	470,414	5,148,043	323,100,000
Age		57.97 (19.22)	54.33 (18.44)	45 (26.15)	56.39 (14.44)	55.25 (18.02)	47.62 (19.04)	49.33 (21.62)	55.68 (16.05)	29.73 (16.87)	62.1 (15.75)	66.22 (14.56)	56.14 (18.64)	62.88 (19.62)	41.45 (16.77)	43.28 (22.83)	47.34 (23.24)	37.9 (median)
Gender	Female	55.78%	67.63%	57.14%	54.48%	52.83%	73.73%	52.90%	86.29%	67.59%	55.36%	44.02%	61.48%	58.72%	72.54%	50.68%	58.86%	51.01%
	Male	44.22%	32.37%	42.86%	45.52%	47.17%	26.27%	47.10%	13.71%	32.41%	44.64%	55.98%	38.52%	41.28%	27.46%	49.32%	41.13%	48.99%
Race	Asian	6.06%	2.11%	6.07%	2.72%	3.68%	6.70%	7.37%	3.25%	5.33%	1.27%	0.88%	2.68%	8.94%	3.13%	4.78%	4.60%	5.67%
	African American	9.05%	3.98%	8.49%	5.76%	7.02%	9.70%	9.34%	15.54%	6.72%	3.33%	3.88%	5.31%	8.51%	18.56%	10.50%	7.37%	13.31%
	Hispanic	8.32%	7.86%	10.38%	9.22%	9.25%	12.94%	16.45%	13.99%	10.13%	4.46%	3.58%	6.75%	14.47%	11.12%	12.52%	9.52%	17.79%
	European	73.34%	82.56%	71.73%	78.76%	76.54%	67.39%	63.13%	64.06%	73.81%	87.61%	88.65%	81.78%	65.53%	63.91%	72.20%	78.51%	61.27%
Education	Below High school	0.32%	0.20%	0.37%	0.36%	0.33%	0.40%	0.60%	0.54%	0.26%	0.09%	0.13%	0.15%	0.43%	0.38%	0.52%	0.29%	16.02%
	High School	22.29%	15.14%	20.17%	23.78%	23.05%	18.34%	20.46%	30.24%	13.59%	15.67%	18.32%	15.17%	31.91%	29.83%	26.40%	18.47%	27.57%
	Below Bachelor	54.53%	54.13%	53.91%	56.46%	54.51%	52.49%	51.87%	54.72%	50.72%	56.54%	57.33%	54.63%	51.06%	55.16%	54.26%	54.20%	45.77%
	Above Bachelor	22.86%	30.52%	25.55%	19.41%	22.12%	28.77%	27.06%	14.49%	35.43%	27.70%	24.22%	30.06%	16.60%	14.62%	18.81%	27.04	10.62%
Home Ownership	Own	90.42%	92.03%	88.59%	89.74%	89.07%	86.74%	88.60%	84.97%	86.86%	94.32%	94.05%	92.50%	91.91%	78.58%	85.06%	89.88%	63.7%
	Rent	9.58%	7.97%	11.41%	10.26%	10.93%	13.26%	11.40%	15.03%	13.14%	5.68%	5.95%	7.50%	8.09%	21.42%	14.94%	10.12%	36.3%
Household Income	<$40k	17.56%	10.30%	15.22%	16.83%	17.57%	14.86%	14.82%	25.94%	9.27%	11.20%	13.93%	10.68%	22.55%	25.46%	19.14%	13.31%	44.82% (<$49k)
	$40k-$49k	5.96%	4.42%	5.75%	6.06%	6.11%	5.83%	5.64%	7.70%	4.15%	4.68%	5.43%	4.40%	8.09%	8.26%	6.95%	5.19%	
	$50k-$59k	7.19%	5.74%	6.72%	7.24%	7.12%	6.59%	6.51%	8.15%	4.67%	6.15%	6.99%	5.68%	9.36%	8.33%	7.57%	6.19%	16.69% ($50k-$74k)
	$60k-$74k	10.93%	9.55%	10.15%	10.90%	10.73%	9.88%	9.95%	11.34%	7.47%	10.31%	11.21%	9.52%	8.09%	10.74%	10.89%	9.69%	
	$75k-$99k	16.77%	16.62%	15.87%	18.01%	16.83%	15.56%	15.87%	15.94%	13.45%	17.69%	18.56%	16.62%	20.00%	15.68%	16.22%	15.95%	12.08%
	>$100k	41.59%	53.38%	46.28%	40.97%	41.64%	47.28%	47.20%	30.93%	60.98%	49.97%	43.89%	53.08%	31.91%	31.53%	39.22%	49.67%	26.41%
Household member	#Adult	1.79 (1.19)	1.98 (1.26)	1.99 (1.24)	1.87 (1.21)	1.85 (1.22)	2.06 (1.3)	2.02 (1.28)	1.75 (1.18)	2.75 (1.49)	1.79 (1.19)	1.65 (1.1)	1.94 (1.26)	1.57 (1.04)	2.06 (1.36)	1.98 (1.25)	2.17 (1.37)	1.94 (0.00)
	#Children	0.26 (0.71)	0.34 (0.79)	0.62 (1.04)	0.24 (0.67)	0.29 (0.74)	0.45 (0.89)	0.48 (0.94)	0.21 (0.62)	0.64 (0.97)	0.21 (0.65)	0.13 (0.52)	0.33 (0.78)	0.21 (0.6)	0.36 (0.78)	0.59 (1.03)	0.48 (0.93)	0.59 (0.00)

PN, prurigo nodularis; AD, atopic dermatitis; PsA, psoriatic arthritis; SLE, systemic lupus erythematosus; HS, hidradenitis suppurativa. This data summarizes data between 2014-2018. The US data come from the US Census Bureau (https://www.census.gov/data/tables).

**Figure 2:**
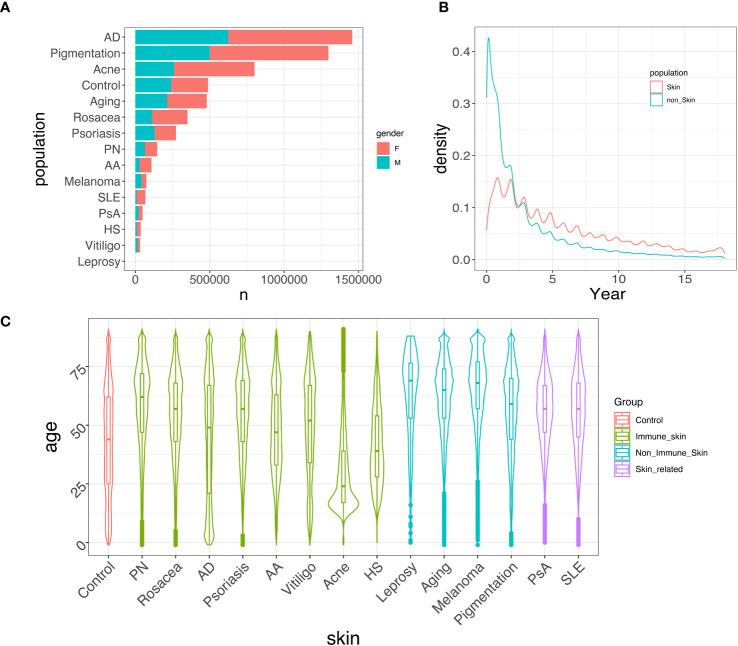
Data summary. **(A)** Gender-specific prevalence of each skin disease/control between 2014-2018. Females generally exhibit a higher prevalence than males in developing immune-mediated skin diseases. **(B)** Distribution of patients’ time in the system, spanning from 2001 to 2018. Most patients stayed in the system for less than 5 years. **(C)** Age distribution of different skin/skin-related diseases/control between 2014-2018. Most skin diseases show a similar age distribution compared to the control group, while acne, AD, and HS tend to have a higher proportion of younger patients.

**Figure 2** provides an overview of the demographic variables in our study. **Figure 2A** displays the prevalence of each skin disease and control categorized by gender. AD, pigmentation, and acne are the most prevalent skin conditions in the CDM data, and their prevalence remains consistent when comparing 2014-2018 records to those from 2001-2005 (**Supplementary Figure 1A**). The gender distributions for different skin conditions also remain consistent. **Figure 2B** presents the density of the time (in years) that patients stay in the CDM system, showing that approximately 60% of the patients stay within a 5-year time span. **Figure 2C** displays the age distribution of the control group and each skin disease group for the period between 2014-2018. This represents the ages of patients with skin-related disorders diagnosis in the system, and not necessarily represent the disease age of onset. Each disease exhibits a unique age distribution compared to the control group. For example, acne patients tend to be younger (29), while AD shows a bimodal pattern in age distribution, which is consistent with previous studies (30). We also observed that the median age for all skin conditions, except for acne, tends to be earlier in the 2001-2005 cohort (**Supplementary Figure 1B**) compared to the 2014-2018 cohort, whereas the age distribution for acne remains consistent over time.

### 3.2 Skin-comorbidity association trends across time

**Figure 3:**
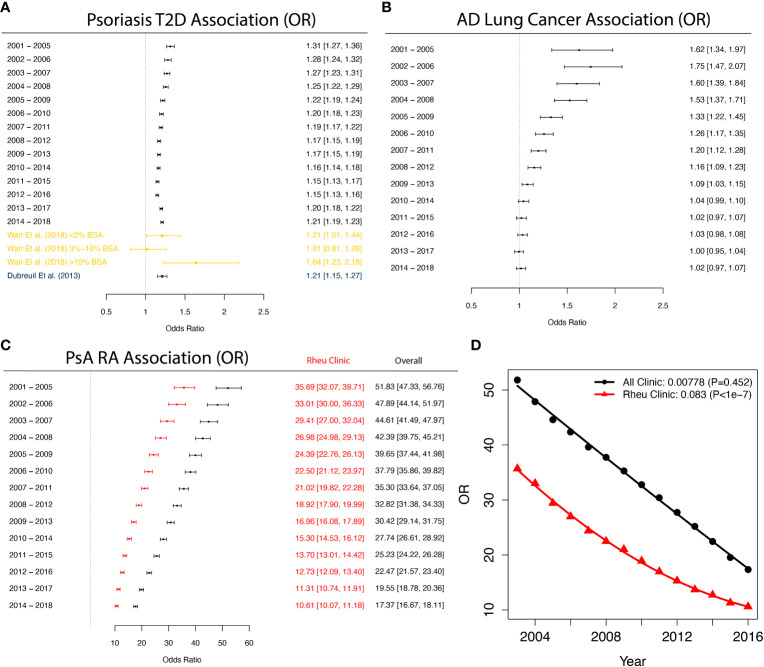
Forest plots of association across a year-to-year period. **(A)** Forest plot illustrating the odds ratio (OR) with confidence intervals (CIs) for the association between psoriasis and type 2 diabetes (T2D) in comparison to non-T2D patients. The OR and CI from this study are shown, along with the corresponding OR and CI from two previous studies for comparison. The findings indicate that the OR estimate from this study aligns with previous results, *but* featuring more precise CI*s*. **(B*)*** Forest plot showcasing the parameter estimate for the OR with CIs of developing atopic dermatitis (AD) in lung cancer patients compared to lung cancer-free patients. The results exhibit a declining trend in the association, which ultimately dissipates. **(C*)*** Forest plot displaying the parameter estimate for the OR with CIs between psoriatic arthritis (PsA) and rheumatoid arthritis (RA) based on all clinics and providers (black) and solely rheumatology clinics (red). The estimated association*s* derived from rheumatology clinics is *weaker* than that from all clinics, with both estimates showing a steady downward trend. This suggests the potential for more precise diagnoses in rheumatology clinics, as well as improved diagnosis accuracy over time in general. **(D)** Regression analysis of PsA vs RA odds ratios based on all clinics and rheumatology clinics, incorporating first-order and second-order time covariates. Estimates and P-values of the second-order time coefficients are shown in the legend. The significant second-order time coefficient from the rheumatology clinic estimate suggests a significant deceleration in the rate of change for ORs, while the rate of change for ORs from all clinics demonstrates a steady decline. For all figures, the control group consists of randomly sampled patients from the general CDM population.

We first investigated the trend of associations between psoriasis and T2D (18, 31), a comorbidity pair that has been extensively studied before. **Figure 3A** provides a summary of adjusted Odds Ratios (ORs) with 95% confidence intervals (CIs) from the logistic regression model. We observed consistent and stable estimated ORs across different time periods, ranging between 1.15 and 1.31. To compare our findings with previous studies (18, 31) on the association between psoriasis and T2D, we included their OR estimates and corresponding 95% CIs. Due to smaller sample sizes, the 95% CIs of these earlier studies are wider compared to our analysis. Although their estimates show some variability, their point estimates for OR align closely with ours, and their 95% CIs encompass most of our estimates.

Furthermore, we explored the association trends of other disease pairs and highlighted notable findings in **Figure 3**. For instance, the association between AD and lung cancer (**Figure 3B**) has transitioned from a significant positive association in the period 2001-2005 (OR: 1.62, 95% CI [1.34-1.97]) to a non-significant association in 2014-2018 (OR: 1.02, 95% CI [0.97-1.07]). While earlier studies from 2005 and 2012 reported positive associations between AD and lung cancer (32, 33), a more recent study in 2020 found that after adjusting for potential mediators such as smoking or smoking-related diseases, this association disappears (34). These findings suggest that improved treatment for AD in recent years or changes in modifying behaviors (such as smoking) may have played a role in reducing the risk of cancer for AD patients. In **Figure 3C**, we observed strong associations between PsA and RA across different years. Since many clinical measures of PsA are adopted from RA (35) and the specific diagnosis of RA and PsA require knowledge from rheumatologists (36), the strong associations may be attributed to miscoding. To explore this further, we conducted separate analyses for patients diagnosed exclusively in rheumatology clinics (red lines in **Figure 3C**), in addition to the analysis based on all clinics or providers (black lines in **Figure 3C**). The associations between PsA and RA from rheumatology clinics consistently exhibit weaker associations compared to the findings from the unrestricted data, while both analyses demonstrate a decreasing trend over time. Although this finding could indicate improving diagnosis accuracy for both rheumatology clinics and other clinics over time, special care is still needed when using medical claims to study disease comorbidities. Additionally, we also observed diminishing differences between the ORs estimated from rheumatology clinics and all clinics (i.e. unrestricted data). We regressed these ORs on both the first-order and second-order time covariates (**Figure 3D**), and found that the second-order term in the regression for all clinics is not significant (*p = 0.452*), indicating that the rate of ORs changing across years remains relatively constant. In contrast, the second-order term in the regression for rheumatology clinics is significant (*p < 1×10^-7^*), suggesting that the changing rate of ORs decreases across years.

### 3.3 Large-scale comorbidity identification

**Figure 4:**
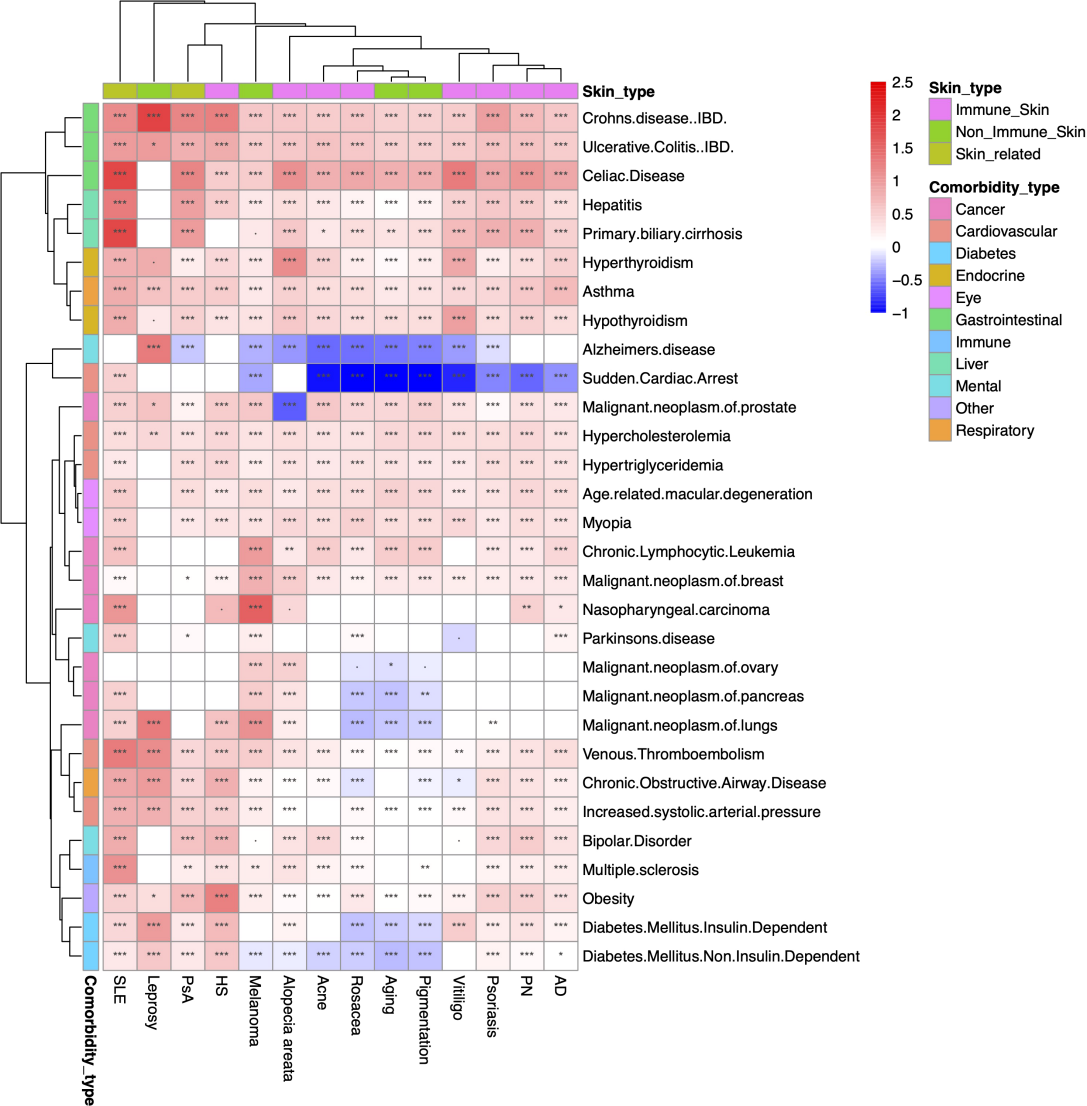
Heatmap of large-scale association results between 2014-2018. Heatmap representation of the associations between overall skin/skin-related conditions and potential comorbidities during the period of 2014-2018. The color intensity reflects the level of odds ratio (OR) association, while asterisks indicate the significance levels (***: P<10^-3^; **: 10^-3^≤P<10^-2^; *: 10^-2^≤P<0.05; ·: 0.05≤P<0.01). The findings suggest that the majority of associations between skin and skin-related conditions and comorbidities are both significant and positive. Particularly notable pairings include Crohn’s disease with leprosy, primary biliary cirrhosis with systemic lupus erythematosus (SLE), and celiac disease with SLE. # The comorbidity analysis does not include rheumatological conditions due to the ambiguity of the phenotyping when using ICD codes and misdiagnosis.

We conducted a large-scale association study to identify the comorbidities for the 14 skin/skin-related conditions using data from the period 2014-2018. We evaluated a total of 420 skin disease-comorbidity pairs by associating the concurrence of these conditions with 30 common human disorders, including respiratory, cancer, mental, immunological, gastrointestinal, cardiovascular, and diabetes conditions (**Figure 4** with detailed association estimates, sample sizes and P-values in **Supplementary Table 2**). For the large-scale comorbidity analysis, we found that most of the skin/skin-related condition-comorbidity associations are significant and positive, with the most prominent associated pairs being Crohn’s disease and leprosy (OR=6.60, 95% CI: 3.09-14.08); primary biliary cirrhosis (PBC) and SLE (OR=6.07, 95% CI: 4.93-7.46); as well as celiac disease (CD) and SLE (OR=6.06, 95% CI: 5.49-6.69). These associations are consistent with previous literature: for instance, different studies have reported overlapping genetic signals between Crohn’s disease and leprosy (37–39). For PBC and SLE, researchers have found the odds of developing PBC is 2.23 (CI: 1.26-3.96) times higher if patients have a family history of SLE (40). A 2016 study estimated the CD and SLE association to be 3.92 in OR (CI 2.55-6.03) (41). Our findings also reveal that patients diagnosed with melanoma have higher rates of being diagnosed with multiple cancers, including ovarian, lung, and prostate cancers. Additionally, we observed that diabetes has either no association or significant negative associations with acne, rosacea, aging, pigmentation, and melanoma. However, among all the skin conditions studied, leprosy patients exhibit the highest odds of co-diagnosis with type I diabetes (OR: 2.71, CI: 1.53-4.80). Our findings align with previous research demonstrating that the incidence of diabetes among leprosy patients is over seven times higher compared to control groups (14.2% vs. 2%) (42). Notably, when compared to the 2001-2005 cohort, the most notable associations remain consistent (**Supplementary Figure 2**), while less associations are observed for multiple cancers.

We presented the effect sizes (in log OR) of all comorbidities for each skin/skin-related condition in the 2014-2018 cohort in **Supplementary Figure 3A**. This highlights that patients with SLE, leprosy, and HS are more susceptible to other comorbid diagnoses. In **Supplementary Figure 3B**, we showed the effect sizes of skin/skin-related conditions within each comorbidity, revealing that celiac disease, Crohn’s disease, and ulcerative colitis have the strongest average associations with the multiple different skin conditions studied in our analysis. We also provided the results for the 2001-2005 cohort in **Supplementary Figure 4**, which generally align with the findings from the 2014-2018 cohort. Additionally, we summarized the 2014-2018 prevalence of the most prevalent comorbidities within controls and patients with skin/skin-related diseases in **Supplementary Table 3**. These results further support that celiac disease is one of the most common comorbidities for patients suffering from skin/skin-related conditions.

## 4 Discussion

Identifying potential comorbidities, particularly those with modest associations, often requires a large sample size for adequate statistical power. Skin conditions, despite being prevalent, are known to have a high percentage of patients who do not seek medical advice, estimated at 73% (43). Consequently, studies in this domain may suffer from limited sample sizes and reduced power to detect weak associations (18, 31). However, leveraging the extensive sample size provided by the claims-based CDM database, we were able to uncover comorbidities even with mild associations. It is worth noting, however, that the CDM database does not include patients insured by Medicaid, which may impact the generalizability of the findings. To validate the CDM dataset, we evaluated the population summary statistics and confirmed their consistency with previous findings regarding overall prevalence, as well as age, ethnicity, and gender distributions. Additionally, we have showcased the well-established link between psoriasis and T2D as a proof-of-concept to further substantiate the validity of the CDM data. We also investigated other skin/skin-related diseases and comorbidities to determine association trends over time. We found that the PsA and RA association decreased dramatically across years. For a long time, PsA was considered to be a variant of RA (44, 45) due to limited knowledge and lack of more specific biomarkers (46). Since the proposition and clinical application of dactylitis as a hallmark and distinct feature of PsA, compared to RA in 1996 (47), and the CASPAR criteria for PsA diagnosis in 2006 (48), our analysis suggests that potential mis-diagnosis is decreasing over time.

We also adopted a different approach to examine the comorbidity: for a particular skin condition (e.g. psoriasis) we randomly selected control patients from the remaining 13 cohorts consisting of patients with different skin conditions. The pipeline and results of this alternative analysis, depicted in **Supplementary Figures 5** and **6**, indicate a generally lower association between psoriasis and T2D compared to the original analysis. This suggests the existence of associations between T2D and other skin conditions within the dataset.

The comorbidity of skin diseases can arise from various mechanisms, and understanding these mechanisms can contribute to a deeper comprehension of disease pathogenesis and enhance diagnostic accuracy. The information on disease co-occurrence would enable researchers to explore shared pathogenesis between these related conditions, thereby advancing the understanding of both conditions. Additionally, comorbidities play a crucial role in dermatological diagnoses, aiding dermatologists in distinguishing different diseases more accurately. The presence of comorbidities can be influenced by treatments administered to patients. In other words, different therapeutic interventions, such as medications, surgeries, or other medical procedures, can have an impact on the occurrence or development of concurrent diseases in individuals with skin conditions. For instance, certain medications used to treat one condition may influence the immune system or physiological processes that could potentially lead to the onset or exacerbation of other diseases. Additionally, the side effects or interactions of medications can also contribute to the development of comorbidities. Moreover, confounding factors such as patients’ lifestyle, quality of life, and living environment can also lead to disease co-occurrence (49). In this analysis, we accounted for potential confounders by adjusting for demographic and socioeconomic variables in the model. Lastly, misdiagnosis can contribute to the observed co-occurrence of two diseases. For example, PsA and RA are susceptible to misdiagnosis, as reported in previous studies (50). In our analysis, we observed a high association between these conditions; however, we also noticed a consistent temporal decrease in this association. This may be attributed to improved diagnostic criteria and a better understanding of disease mechanisms. Nevertheless, it is important to note that our association analysis does not completely eliminate the potential of misdiagnosis. We recommend that future systematic studies consider employing machine learning methods to correct phenotyping and address misdiagnosis as a preliminary step (51, 52).

## Data availability statement

The data analyzed in this study is subject to the following licenses/restrictions: The data analyzed in this study was obtained from Optum’s de-identified Clinformatics^®^ Data Mart Database, and cannot be directly shared to the public. Requests to access these datasets should be directed to Optum, connected@optum.com.

## Author contributions

QL: Data curation, Formal Analysis, Investigation, Methodology, Software, Writing – original draft. MP: Data curation, Methodology, Supervision, Writing – review & editing. SS: Data curation, Writing – review & editing. JK: Supervision, Writing – review & editing. JMK: Writing – review & editing. JG: Writing – review & editing. KH: Methodology, Supervision, Writing – review & editing. LT: Conceptualization, Funding acquisition, Methodology, Project administration, Supervision, Writing – review & editing.
