# Supplementary material for: Large-scale epidemiological analysis of common skin diseases to identify shared and unique comorbidities and demographic factors
Source: Front Immunol. 2024 Jan 8;14:1309549. doi: 10.3389/fimmu.2023.1309549 (PMC10800546; doi:10.3389/fimmu.2023.1309549)
Supplement: Supplementary file 1 [file DataSheet_1.docx]

**Supplemental materials**

**Supplemental Figure1. Data summary.** **a.** Gender-specific prevalence of each skin disease/control between 2001-2005. The results are consistent with 2014-2018 data. **b.** Age distribution of different skin diseases/control between 2001-2005. Consistent with 2014-2018 results, most skin diseases show a similar age distribution compared to the control group, while acne, AD, and HS tend to have a higher proportion of younger patients.

**Supplemental Figure 2.** **Heatmap of large-scale association results between 2001-2005.** Heatmap representation of the associations between overall skin/skin-related conditions and potential comorbidities during the period of 2001-2005. The color intensity reflects the level of odds ratio (OR) association, while asterisks indicate the significance levels (*** : P<10^-3^; ** : 10^-3^≤P<10^-2^; * : 10^-2^≤P<0.05; ⋅ : 0.05≤P<0.01). When compared to the results from 2014-2018, the most notable associations remain consistent. However, an increase in associations with multiple cancers has been observed. #The comorbidity analysis does not include rheumatological conditions due to the ambiguity of the phenotyping when using ICD codes and misdiagnosis.

**Supplemental Figure 3. Marginal association plots.** The left figure displays box plots illustrating the distribution of all potential comorbidity associations for each skin condition during the period of 2014-2018. The right figure displays box plots representing the distribution of all skin condition associations for each comorbidity during the same time frame. The findings suggest that systemic lupus erythematosus (SLE), leprosy, and hidradenitis suppurativa (HS) have the strongest average association with general comorbidities. Conversely, celiac disease, Crohn's disease, and ulcerative colitis demonstrate the highest average association with skin and skin-related conditions.

**Supplemental Figure 4.** **Marginal association plots.** The left figure displays box plots illustrating the distribution of all potential comorbidity associations for each skin condition during the period of 2001-2005. The right figure displays box plots representing the distribution of all skin condition associations for each comorbidity during the same time frame. The skin and skin-related conditions and comorbidities with the highest average associations notably coincide with the findings from the 2014-2018 study period.

**Supplemental Figure 5. Data preprocessing and model fitting workflow for the alternative analysis.** The flowchart illustrates the selection process for patients with skin-related conditions and the control group. Patients with the 14 skin-related conditions are initially extracted. Quality control steps are applied to remove patients with incomplete records. The subsequent statistical analyses involve comparing patients having one of the extracted skin conditions against the other 13 skin-conditioned patients as the control group.

**Supplemental Figure 6. Forest plots of association** **across a year-to-year period. a.** Forest plot illustrating the odds ratio (OR) with confidence intervals (CIs) for the association between psoriasis and type 2 diabetes (T2D) in comparison to non-T2D patients. The OR and CI from this study are shown, along with the corresponding OR and CI from two previous studies for comparison. The control group consists of the other 13 skin/skin-related disordered patients besides psoriasis. **b.** Forest plot showcasing the parameter estimate for the OR with CIs of developing atopic dermatitis (AD) in lung cancer patients compared to lung cancer-free patients. The control group consists of the other 13 skin/skin-related disordered patients besides AD.

**Supplemental Table 1. Condition definition and ICD9/10 codes**

| **­­­Condition type I** | **Condition type II** | **Condition** | **ICD9/ICD10** |
| --- | --- | --- | --- |
| **Skin/skin-related** | **Immune-mediated skin disease** | **Acne** | 706 706.1 L70 L70.0 L70.1 L70.2 L70.3 L70.4 L70.5 L70.8 L70.9 |
|  |  | **Rosacea** | 695.3 L71 L71.0 L71.1 L71.8 L71.9 |
|  |  | **Alopecia areata** | 704 704.01 704.09 L63 L63.0 L63.1 L63.2 L63.8 L63.9 |
|  |  | **Vitiligo** | 709.01 L80 H02.73 H02.731 H02.732 H02.733 H02.734 H02.735 H02.736 H02.739 |
|  |  | **Psoriasis** | 696.1 696.8 L40 L40.0 L40.1 L40.2 L40.3 L40.4 L40.8 L40.9 696 L40.5 L40.50 L40.51 L40.52 L40.53 L40.54 L40.59 |
|  |  | **Atopic dermatitis** | 691.8 692.9 L20.0 L20.8 L20.81 L20.82 L20.83 L20.84 L20.89 L20.9 L30.9 |
|  |  | **Hidradenitis suppurativa** | 705.83 L73.2 |
|  |  | **Prurigo nodularis** | 698.2 L28.1 L28.0 L28.2 698.3 |
|  | **Non-immune-mediated skin disease** | **Aging** | 692.74 L57.8 |
|  |  | **Leprosy** | 30 30.1 30.2 30.3 30.8 30.9 A30 A30.0 A30.1 A30.2 A30.3 A30.4 A30.5 A30.8 A30.9 |
|  |  | **Pigmentation** | 709 709.09 L81.0 L81.1 L81.2 L81.3 L81.4 L81.5 L81.6 L81.7 L81.8 L81.9 |
|  |  | **Melanoma** | 172 172.1 172.2 172.3 172.4 172.5 172.6 172.7 172.8 172.9 C43 C43.0 C43.1 C43.10 C43.11 C43.12 C43.2 C43.20 C43.21 C43.22 C43.3 C43.30 C43.31 C43.39 C43.4 C43.5 C43.51 C43.52 C43.59 C43.6 C43.60 C43.61 C43.62 C43.70 C43.7 C43.71 C43.72 C43.8 C43.9 |
|  | **Skin-related disorders** | **Systemic lupus erythematosus** | 710 M32.1 M32.10 M32.11 M32.12 M32.13 M32.14 M32.15 M32.19 M32.8 M32.9 |
|  |  | **Psoriatic arthritis** | 696 L40.5 L40.50 L40.51 L40.52 L40.53 L40.54 L40.59 |
| **Potential comorbidities** | **Eye** | **Age related macular degeneration** | 362.5 362.51 362.52 H35.30 H35.31 H35.311 H35.3110 H35.3111 H35.3112 H35.3113 H35.3114 H35.312 H35.3120 H35.3121 H35.3122 H35.3123 H35.3124 H35.313 H35.3130 H35.3131 H35.3132 H35.3133 H35.3134 H35.319 H35.3190 H35.3191 H35.3192 H35.3193 H35.3194 H35.32 H35.321 H35.3210 H35.3211 H35.3212 H35.3213 H35.322 H35.3220 H35.3221 H35.3222 H35.3223 H35.323 H35.3230 H35.3231 H35.3232 H35.3233 H35.329 H35.3290 H35.3291 H35.3292 H35.3293 |
|  |  | **Myopia** | 367.1 360.21 H44.2 H44.20 H44.21 H44.22 H44.23 H44.2A H44.2A1 H44.2A2 H44.2A3 H44.2A9 H44.2B H44.2B1 H44.2B2 H44.2B3 H44.2B9 H44.2C H44.2C1 H44.2C2 H44.2C3 H44.2C9 H44.2D H44.2D1 H44.2D2 H44.2D3 H44.2D9 H44.2E H44.2E1 H44.2E2 H44.2E3 H44.2E9 H52.10 H52.11 H52.12 H52.13 |
|  | **Mental** | **Alzheimer’s disease** | 331 G30 G30.0 G30.1 G30.8 G30.9 |
|  |  | **Bipolar disorder** | 269 296 296.01 296.02 296.03 296.04 296.05 296.06 296.1 296.1 296.11 296.12 296.13 296.14 296.15 296.16 296.4 296.4 296.41 296.42 296.43 296.44 296.45 296.46 296.5 296.5 296.51 296.52 296.53 296.54 296.55 296.56 296.6 296.6 296.61 296.62 296.63 296.64 296.65 296.66 296.7 296.8 296.81 296.89 F31 F31.0 F31.1 F31.10 F31.11 F31.12 F31.13 F31.2 F31.3 F31.30 F31.31 F31.32 F31.4 F31.5 F31.6 F31.60 F31.61 F31.62 F31.63 F31.64 F31.7 F31.70 F31.71 F31.72 F31.73 F31.74 F31.75 F31.76 F31.77 F31.78 F31.8 F31.81 F31.89 F31.9 |
|  |  | **Parkinson’s disease** | 332 G20 |
|  | **Respiratory** | **Asthma** | 493 493.01 493.02 493.1 493.1 493.11 493.12 493.2 493.2 492.21 493.22 493.8 493.81 493.82 493.9 493.9 493.91 493.92 J45 J45.2 J45.20 J45.21 J45.22 J45.3 J45.30 J45.31 J45.32 J45.4 J45.40 J45.41 J45.42 J45.5 J45.50 J45.51 J45.52 J45.9 J45.90 J45.901 J45.902 J45.909 J45.99 J45.990 J45.991 J45.998 |
|  |  | **Chronic obstructive airway disease** | 491 491.1 491.2 491.2 491.21 491.22 491.8 491.9 492 492 492.8 496 J41 J41.0 J41.1 J41.8 J42 J43 J43.0 J43.1 J43.2 J43.8 J43.9 J44 J44.0 J44.1 J44.9 |
|  | **Gastrointestinal** | **Celiac disease** | 579 K90.0 |
|  |  | **Crohns disease (IBD)** | 555 555.1 555.2 555.9 K50 K50.0 K50.00 K50.01 K50.011 K50.012 K50.013 K50.014 K50.018 K50.019 K50.1 K50.10 K50.11 K50.111 K50.112 K50.113 K50.114 K50.118 K50.119 K50.8 K50.80 K50.81 K50.811 K50.812 K50.813 K50.814 K50.818 K50.819 K50.9 K50.90 K50.91 K50.911 K50.912 K50.913 K50.914 K50.918 K50.919 |
|  |  | **Ulcerative colitis (IBD)** | 556 556.1 556.2 556.3 556.4 556.5 556.6 556.8 556.9 K51 K51.0 K51.00 K51.01 K51.011 K51.012 K51.013 K51.014 K51.018 K51.019 K51.2 K51.20 K51.21 K51.211 K51.212 K51.213 K51.214 K51.218 K51.219 K51.3 K51.30 K51.31 K51.311 K51.312 K51.313 K51.314 K51.318 K51.319 K51.4 K51.40 K51.41 K51.411 K51.412 K51.413 K51.414 K51.418 K51.419 K51.5 K51.50 K51.51 K51.511 K51.512 K51.513 K51.514 K51.518 K51.519 K51.8 K51.80 K51.81 K51.811 K51.812 K51.813 K51.814 K51.818 K51.819 K51.9 K51.90 K51.91 K51.911 K51.912 K51.913 K51.914 K51.918 K51.919 |
|  | **Cancer** | **Chronic lymphocytic leukemia** | 204.1 204.11 204.12 C91.1 C91.10 C91.11 C91.12 C91.3 C91.30 C91.31 C91.32 C91.6 C91.60 C91.61 C91.62 |
|  |  | **Malignant neoplasm of breast** | 174 174.1 174.2 174.3 174.4 174.5 174.6 174.8 174.9 175 175 175.9 C50  C50.0  C50.01  C50.011 C50.012 C50.019 C50.02 C50.021 C50.022 C50.029 C50.1 C50.11 C50.111 C50.112 C50.119 C50.12 C50.121 C50.122 C50.129 C50.2 C50.21 C50.211 C50.212 C50.219 C50.22 C50.221 C50.222 C50.229 C50.3 C50.31 C50.311 C50.312 C50.319 C50.32 C50.321 C50.322 C50.329 C50.4 C50.41 C50.411 C50.412 C50.419 C50.42 C50.421 C50.422 C50.429 C50.5 C50.51 C50.511 C50.512 C50.519 C50.52 C50.521 C50.522 C50.529 C50.6 C50.61 C50.611 C50.612 C50.619 C50.62 C50.621 C50.622 C50.629 C50.8 C50.81 C50.811 C50.812 C50.819 C50.82 C50.821 C50.822 C50.829 C50.9 C50.91 C50.911 C50.912 C50.919 C50.92 C50.921 C50.922 C50.929 |
|  |  | **Malignant neoplasm of lungs** | 162.3 162.4 162.5 162.8 162.9 209.21 C34.1 C34.10 C34.11 C34.12 C34.2 C34.3 C34.30 C34.31 C34.32 C34.8 C34.80 C34.81 C34.82 C34.9 C34.90 C34.91 C34.92 C7A.090 |
|  |  | **Malignant neoplasm of ovary** | 183 C56 C56.1 C56.2 C56.9 |
|  |  | **Malignant neoplasm of pancreas** | 157 157.1 157.2 157.3 157.4 157.8 157.9 C25 C25.0 C25.1 C25.2 C25.3 C25.4 C25.7 C25.8 C25.9 |
|  |  | **Malignant neoplasm of prostate** | 185 C61 |
|  |  | **Nasopharyngeal carcinoma** | 147 147.1 147.2 147.3 147.8 147.9 C11 C11.0 C11.1 C11.2 C11.3 C11.8 C11.9 |
|  | **Diabetes** | **Diabetes mellitus/ insulin-dependent** | 250.01 250.03 250.11 250.13 250.21 250.23 250.31 250.33 250.41 250.43 250.51 250.53 250.61 250.63 250.71 250.73 250.81 250.83 250.91 250.93 E10.1 E10.10 E10.11 E10.2 E10.21 E10.22 E10.29 E10.3 E10.31 E10.311 E10.319 E10.32 E10.321 E10.3211 E10.3212 E10.3213 E10.3219 E10.329 E10.3291 E10.3292 E10.3293 E10.3299 E10.33 E10.331 E10.3311 E10.3312 E10.3313 E10.3319 E10.339 E10.3391 E10.3392 E10.3393 E10.3399 E10.34 E10.341 E10.3411 E10.3412 E10.3413 E10.3419 E10.349 E10.3491 E10.3492 E10.3493 E10.3499 E10.35 E10.351 E10.3511 E10.3512 E10.3513 E10.3519 E10.352 E10.3521 E10.3522 E10.3523 E10.3529 E10.353 E10.3531 E10.3532 E10.3533 E10.3539 E10.354 E10.3541 E10.3542 E10.3543 E10.3549 E10.355 E10.3551 E10.3552 E10.3553 E10.3559 E10.359 E10.3591 E10.3592 E10.3593 E10.3599 E10.36 E10.37 E10.37X1 E10.37X2 E10.37X3 E10.37X9 E10.39 E10.4 E10.40 E10.41 E10.42 E10.43 E10.44 E10.49 E10.5 E10.51 E10.52 E10.59 E10.6 E10.61 E10.610 E10.618 E10.62 E10.620 E10.621 E10.622 E10.628 E10.63 E10.630 E10.638 E10.64 E10.641 E10.649 E10.65 E10.69 E10.8 E10.9 |
|  |  | **Diabetes mellitus/ non-insulin-dependent** | 250 250.02 250.1 250.12 250.2 250.22 250.3 250.32 250.4 250.42 250.5 250.52 250.6 250.62 250.7 250.72 250.8 250.82 250.9 250.92 E11 E11.0 E11.00 E11.01 E11.1 E11.10 E11.11 E11.2 E11.21 E11.22 E11.29 E11.3 E11.31 E11.311 E11.319 E11.32 E11.321 E11.3211 E11.3212 E11.3213 E11.3219 E11.329 E11.3291 E11.3292 E11.3293 E11.3299 E11.33 E11.331 E11.3311 E11.3312 E11.3313 E11.3319 E11.339 E11.3391 E11.3392 E11.3393 E11.3399 E11.34 E11.341 E11.3411 E11.3412 E11.3413 E11.3419 E11.349 E11.3491 E11.3492 E11.3493 E11.3499 E11.35 E11.351 E11.3511 E11.3512 E11.3513 E11.3519 E11.352 E11.3521 E11.3522 E11.3523 E11.3529 E11.353 E11.3541 E11.3542 E11.3543 E11.3549 E11.354 E11.3541 E11.3542 E11.3543 E11.3549 E11.355 E11.3551 E11.3552 E11.3553 E11.3559 E11.359 E11.3591 E11.3592 E11.3593 E11.3599 E11.36 E11.37 E11.37X1 E11.37X2 E11.37X3 E11.37X9 E11.39 E11.4 E11.40 E11.41 E11.42 E11.43 E11.44 E11.49 E11.5 E11.51 E11.52 E11.59 E11.6 E11.61 E11.610 E11.618 E11.62 E11.620 E11.621 E11.622 E11.628 E11.63 T E11.630 E11.638 E11.64 E11.641 E11.649 E11.65 E11.69 E11.8 E11.9 |
|  | **Liver** | **Hepatitis** | 70 70.1 70.2 70.2 70.21 70.22 70.23 70.3 70.3 70.31 70.32 70.33 70.4 70.41 70.42 70.43 70.44 70.49 70.5 70.51 70.52 70.53 70.54 70.59 70.6 70.7 70.7 70.71 70.9 72.71 91.62 130.5 570 571.1 571.4 571.4 571.41 571.42 571.49 573.1 573.2 573.3 A51.45 B00.81 B15 B15.0 B15.9 B16 B16.0 B16.1 B16.2 B16.9 B17 B17.0 B17.1 B17.10 B17.11 B17.2 B17.8 B17.9 B18 B18.0 B18.1 B18.2 B18.8 B18.9 B19 B19.0 B19.1 B19.10 B19.11 B19.2 B19.20 B19.21 B19.9 B25.1 B26.81 B58.1 K70.1 K70.10 K70.11 K71.0 K71.2 K71.3 K71.4 K71.5 K71.50 K71.51 K71.6 K72 K72.0 K72.00 K72.01 K72.1 K72.10 K72.11 K72.9 K72.90 K72.91 K73 K73.0 K73.1 K73.2 K73.8 K73.9 K75.2 K75.3 K75.4 K75.8 K75.81 K75.89 K75.9 |
|  |  | **Primary biliary cirrhosis** | 571.6 K74.3 |
|  | **Cardiovascular** | **Hypercholesterolemia** | 272 272.2 E78.0 E78.00 E78.01 E78.2 |
|  |  | **Hypertriglyceridemia** | 272.1 E78.1 |
|  |  | **Increased systolic arterial pressure** | 401 401.1 401.9 796.2 I10 R03.0 |
|  |  | **Sudden cardiac arrest** | 427.5 I46.2 I46.8 I46.9 |
|  |  | **Venous thromboembolism** | 452 453 453 453.1 453.2 453.3 453.4 453.4 453.41 453.42 453.5 453.5 453.51 453.52 453.6 453.7 453.71 453.72 453.73 453.74 453.75 453.76 453.77 453.79 453.8 453.81 453.82 453.83 453.84 453.85 453.86 453.87 453.89 453.9 I81 I82 I82.0 I82.1 I82.2 I82.21 I82.210 I82.211 I82.22 I82.220 I82.221 I82.29 I82.290 I82.291 I82.3 I82.4 I82.40 I82.401 I82.402 I82.403 I82.409 I82.41 I82.411 I82.412 I82.413 I82.419 I82.42 I82.421 I82.422 I82.423 I82.429 I82.43 I82.431 I82.432 I82.433 I82.439 I82.44 I82.441 I82.442 I82.443 I82.449 I82.49 I82.491 I82.492 I82.493 I82.499 I82.4Y I82.4Y1 I82.4Y2 I82.4Y3 I82.4Y9 I82.4Z I82.4Z1 I82.4Z2 I82.4Z3 I82.4Z9 I82.5 I82.50 I82.501 I82.502 I82.503 I82.509 I82.51 I82.511 I82.512 I82.513 I82.519 I82.52 I82.521 I82.522 I82.523 I82.529 I82.53 I82.531 I82.532 I82.533 I82.539 I82.54 I82.541 I82.542 I82.543 I82.549 I82.59 I82.591 I82.592 I82.593 I82.599 I82.5Y I82.5Y1 I82.5Y2 I82.5Y3 I82.5Y9 I82.5Z I82.5Z1 I82.5Z2 I82.5Z3 I82.5Z9 I82.6 I82.60 I82.601 I82.602 I82.603 I82.609 I82.61 I82.611 I82.612 I82.613 I82.619 I82.62 I82.621 I82.622 I82.623 I82.629 I82.7 I82.70 I82.701 I82.702 I82.703 I82.709 I82.71 I82.711 I82.712 I82.713 I82.719 I82.72 I82.721 I82.722 I82.723 I82.729 I82.A I82.A1 I82.A11 I82.A12 I82.A13 I82.A19 I82.A2 I82.A21 I82.A22 I82.A23 I82.A29 I82.B I82.B1 I82.B11 I82.B12 I82.B13 I82.B19 I82.B2 I82.B21 I82.B22 I82.B23 I82.B29 I82.C I82.C1 I82.C11 I82.C12 I82.C13 I82.C19 I82.C2 I82.C21 I82.C22 I82.C23 I82.C29 I82.8 I82.81 I82.811 I82.812 I82.813 I82.819 I82.89 I82.890 I82.891 I82.9 I82.90 I82.91 |
|  | **Endocrine** | **Hyperthyroidism** | 242 242.01 242.1 242.1 242.11 242.2 242.2 242.21 242.3 242.3 242.31 242.4 242.4 242.41 242.8 242.8 242.81 242.9 242.9 242.91 E05.0 E05.00 E05.01 E05.1 E05.10 E05.11 E05.2 E05.20 E05.21 E05.3 E05.30 E05.31 E05.8 E05.80 E05.81 E05.9 E05.90 E05.91 |
|  |  | **Hypothyroidism** | 243 245.2 E06.3 E03 E03.0 E03.1 E03.2 E03.3 E03.4 E03.5 E03.8 E03.9 |
|  | **Immune** | **Multiple sclerosis** | 340 G35 |
|  | **Other** | **Obesity** | 278 278.01 278.03 V85.3 V85.30 V85.31 V85.32 V85.33 V85.34 V85.35 V85.36 V85.37 V85.38 V85.39 V85.4 V85.41 V85.42 V85.43 V85.44 V85.45 V85.54 E66.01 E66.09 E66.1 E66.2 E66.8 E66.9 Z68.3 Z68.30 Z68.31 Z68.32 Z68.33 Z68.34 Z68.35 Z68.36 Z68.37 Z68.38 Z68.39 Z68.4 Z68.41 Z68.42 Z68.43 Z68.44 Z68.45 Z68.54 |

**Supplemental Table 2. Large-scale association estimates, P-values and sample-sizes between 2014-2018**

|  | Prurigo nodularis (617,210) | Rosacea (821,440) | Atopic dermatitis (1,928,831) | Psoriatic arthropathy (518,655) | Psoriasis (743,327) | Alopecia areata (578,876) | Vitiligo (502,328) | Lupus erythematosus systemic (538,132) | Acne (1,271,564) | Aging (950,829) | Melanoma (544,342) | Pigmentation (1,768,363) | Leprosy (470,649) | Hidradenitis suppurativa (506,778) |
| --- | --- | --- | --- | --- | --- | --- | --- | --- | --- | --- | --- | --- | --- | --- |
| Age related macular degeneration | 1.58 (<10^-16) | 1.5 (<10^-16) | 1.45 (<10^-16) | 1.43 (<10^-16) | 1.4 (<10^-16) | 1.35 (<10^-16) | 1.32 (10^-15.46) | 1.71 (<10^-16) | 1.48 (<10^-16) | 1.59 (<10^-16) | 1.48 (<10^-16) | 1.53 (<10^-16) | 1.15 (0.6) | 1.35 (10^-8.79) |
| Alzheimer’s disease | 1.04 (0.15) | 0.59 (<10^-16) | 0.98 (0.21) | 0.81 (­10^-3.76) | 0.87 (10^-7.58) | 0.67 (<10^-16) | 0.68 (10^-7.78) | 1.04 (0.37) | 0.57 (<10^-16) | 0.59 (<10^-16) | 0.74 (<10^-16) | 0.62 (<10^-16) | 3.59 (10^-6.33) | 0.88 (0.2) |
| Asthma | 1.8 (<10^-16) | 1.44 (<10^-16) | 1.96 (<10^-16) | 1.74 (<10^-16) | 1.52 (<10^-16) | 1.63 (<10^-16) | 1.52 (<10^-16) | 2.28 (<10^-16) | 1.49 (<10^-16) | 1.33 (<10^-16) | 1.31 (<10^-16) | 1.38 (<10^-16) | 1.91 (10^-3.47) | 1.72 (<10^-16) |
| Bipolar disorder | 1.68 (<10^-16) | 1.15 (10^-10.09) | 1.43 (<10^-16) | 1.86 (<10^-16) | 1.52 (<10^-16) | 1.42 (<10^-16) | 1.09 (0.1) | 2.31 (<10^-16) | 1.51 (<10^-16) | 1.01 (0.78) | 1.08 (0.06) | 1.02 (0.15) | 0.73 (0.65) | 2.1 (<10^-16) |
| Celiac disease | 2.78 (<10^-16) | 2.44 (<10^-16) | 2.39 (<10^-16) | 3.39 (<10^-16) | 2.48 (<10^-16) | 3.02 (<10^-16) | 3.74 (<10^-16) | 6.06 (<10^-16) | 2.23 (<10^-16) | 2.2 (<10^-16) | 1.69 (10^-11.93) | 2.19 (<10^-16) | 1.75 (0.58) | 1.68 (10^-8.11) |
| Chronic lymphocytic leukemia | 1.32 (10^-4.31) | 1.33 (10^-5.62) | 1.54 (<10^-16) | 1.04 (0.74) | 1.31 (10^-5.28) | 1.35 (10^-2.74) | 1 (0.98) | 1.92 (10^-11.32) | 1.72 (10^-14.08) | 1.74 (<10^-16) | 2.66 (<10^-16) | 1.73 (<10^-16) | 0 (0.97) | 1.32 (0.15) |
| Chronic obstructive airway disease | 1.39 (<10^-16) | 0.89 (<10^-16) | 1.29 (<10^-16) | 1.53 (<10^-16) | 1.46 (<10^-16) | 1.09 (10^-7.18) | 0.94 (0.01) | 2.43 (<10^-16) | 1.13 (<10^-16) | 0.99 (0.22) | 1.17 (<10^-16) | 0.96 (10^-6.74) | 2.68 (10^-8.75) | 2.2 (<10^-16) |
| Crohn’s disease (IBD) | 1.99 (<10^-16) | 1.84 (<10^-16) | 1.8 (<10^-16) | 3.31 (<10^-16) | 2.62 (<10^-16) | 1.77 (<10^-16) | 1.69 (10^-11.63) | 3.2 (<10^-16) | 1.67 (<10^-16) | 1.59 (<10^-16) | 1.76 (<10^-16) | 1.65 (<10^-16) | 6.6 (10^-5.97) | 3.56 (<10^-16) |
| Diabetes mellitus insulin-dependent | 1.39 (<10^-16) | 0.8 (<10^-16) | 1.21 (<10^-16) | 1.32 (10^-13.66) | 1.25 (<10^-16) | 1.17 (10^-5.86) | 1.69 (<10^-16) | 1.56 (<10^-16) | 0.97 (0.18) | 0.82 (<10^-16) | 1.04 (0.25) | 0.87 (10^-15.48) | 2.71 (10^-3.22) | 1.94 (<10^-16) |
| Diabetes mellitus non-insulin-dependent | 1.11 (<10^-16) | 0.83 (<10^-16) | 1.01 (0.02) | 1.32 (<10^-16) | 1.21 (<10^-16) | 0.94 (10^-8.32) | 1.01 (0.41) | 1.35 (<10^-16) | 0.85 (<10^-16) | 0.77 (<10^-16) | 0.91 (<10^-16) | 0.79 (<10^-16) | 1.82 (10^-4.09) | 1.78 (<10^-16) |
| Hepatitis | 1.68 (<10^-16) | 1.19 (<10^-16) | 1.45 (<10^-16) | 2.55 (<10^-16) | 1.78 (<10^-16) | 1.43 (<10^-16) | 1.6 (<10^-16) | 3.76 (<10^-16) | 1.33 (<10^-16) | 1.09 (10^-5.4) | 1.34 (<10^-16) | 1.17 (<10^-16) | 1.36 (0.42) | 1.69 (<10^-16) |
| Hypercholesterolemia | 1.56 (<10^-16) | 1.42 (<10^-16) | 1.39 (<10^-16) | 1.42 (<10^-16) | 1.45 (<10^-16) | 1.43 (<10^-16) | 1.47 (<10^-16) | 1.47 (<10^-16) | 1.42 (<10^-16) | 1.52 (<10^-16) | 1.42 (<10^-16) | 1.51 (<10^-16) | 1.53 (10^-2.4) | 1.53 (<10^-16) |
| Hyperthyroidism | 1.45 (<10^-16) | 1.25 (10^-9.96) | 1.58 (<10^-16) | 1.28 (10^-4.25) | 1.28 (10^-11.72) | 3.2 (<10^-16) | 2.42 (<10^-16) | 2.19 (<10^-16) | 1.64 (<10^-16) | 1.15 (10^-4.77) | 1.31 (10^-6.48) | 1.27 (<10^-16) | 2.34 (0.09) | 1.54 (10^-8.54) |
| Hypertriglyceridemia | 1.49 (<10^-16) | 1.39 (<10^-16) | 1.39 (<10^-16) | 1.46 (<10^-16) | 1.44 (<10^-16) | 1.42 (<10^-16) | 1.32 (10^-12.53) | 1.34 (<10^-16) | 1.35 (<10^-16) | 1.36 (<10^-16) | 1.28 (<10^-16) | 1.35 (<10^-16) | 0.81 (0.65) | 1.54 (<10^-16) |
| Hypothyroidism | 1.59 (<10^-16) | 1.5 (<10^-16) | 1.43 (<10^-16) | 1.64 (<10^-16) | 1.46 (<10^-16) | 1.83 (<10^-16) | 2.58 (<10^-16) | 2.36 (<10^-16) | 1.55 (<10^-16) | 1.46 (<10^-16) | 1.39 (<10^-16) | 1.49 (<10^-16) | 1.36 (0.07) | 1.4 (<10^-16) |
| Increased systolic arterial pressure | 1.36 (<10^-16) | 1.11 (<10^-16) | 1.25 (<10^-16) | 1.64 (<10^-16) | 1.38 (<10^-16) | 1.11 (<10^-16) | 1.11 (10^-10.79) | 2.17 (<10^-16) | 1 (0.93) | 1.05 (10^-15.7) | 1.24 (<10^-16) | 1.04 (<10^-16) | 2.15 (10^-4.25) | 1.64 (<10^-16) |
| Malignant neoplasm of breast | 1.3 (<10^-16) | 1.18 (<10^-16) | 1.31 (<10^-16) | 1.09 (0.02) | 1.23 (<10^-16) | 1.69 (<10^-16) | 1.28 (10^-7.58) | 1.15 (10^-7.2) | 1.31 (<10^-16) | 1.33 (<10^-16) | 2.16 (<10^-16) | 1.35 (<10^-16) | 0.96 (0.92) | 1.19 (10^-3.67) |
| Malignant neoplasm of lungs | 0.99 (0.69) | 0.77 (10^-14.11) | 1.02 (0.46) | 1.02 (0.74) | 1.09 (10^-2.41) | 1.23 (10^-4.94) | 0.9 (0.2) | 1.6 (<10^-16) | 1.05 (0.26) | 0.81 (10^-12.31) | 2.99 (<10^-16) | 0.83 (10^-12.59) | 3.75 (10^-4.23) | 1.86 (10^-14.92) |
| Malignant neoplasm of ovary | 0.97 (0.7) | 0.89 (0.06) | 1.05 (0.3) | 0.97 (0.82) | 0.96 (0.5) | 1.62 (10^-10.77) | 0.85 (0.32) | 1.01 (0.93) | 0.98 (0.72) | 0.87 (0.02) | 1.7 (10^-9.94) | 0.92 (0.1) | 0 (0.96) | 1.03 (0.84) |
| Malignant neoplasm of pancreas | 0.96 (0.55) | 0.8 (10^-3.07) | 1.02 (0.62) | 0.89 (0.35) | 0.97 (0.66) | 1.41 (10^-3.91) | 0.87 (0.39) | 1.66 (10^-7.45) | 1.07 (0.36) | 0.8 (10^-4.17) | 1.71 (10^-12.71) | 0.87 (10^-2.34) | 1.56 (0.66) | 1.22 (0.26) |
| Malignant neoplasm of prostate | 1.38 (<10^-16) | 1.57 (<10^-16) | 1.3 (<10^-16) | 1.17 (10^-4.6) | 1.13 (10^-8.48) | 0.54 (<10^-16) | 1.37 (10^-12.75) | 1.67 (<10^-16) | 1.8 (<10^-16) | 1.57 (<10^-16) | 1.78 (<10^-16) | 1.6 (<10^-16) | 1.9 (0.05) | 1.68 (10^-14.26) |
| Multiple sclerosis | 1.26 (10^-5.77) | 1.14 (10^-3.19) | 1.28 (10^-15.46) | 1.24 (10^-2.93) | 1.18 (10^-4.67) | 1.38 (10^-9.79) | 0.95 (0.59) | 3.01 (<10^-16) | 1.2 (10^-6.02) | 1.04 (0.36) | 1.23 (10^-2.88) | 1.09 (10^-2.4) | 2.39 (0.22) | 1.38 (10^-4.47) |
| Myopia | 1.47 (<10^-16) | 1.59 (<10^-16) | 1.43 (<10^-16) | 1.33 (<10^-16) | 1.35 (<10^-16) | 1.57 (<10^-16) | 1.51 (<10^-16) | 1.66 (<10^-16) | 1.5 (<10^-16) | 1.47 (<10^-16) | 1.39 (<10^-16) | 1.48 (<10^-16) | 1.46 (0.14) | 1.36 (<10^-16) |
| Nasopharyngeal carcinoma | 1.66 (10^-2.42) | 0.86 (0.43) | 1.35 (0.02) | 1.01 (0.98) | 1.03 (0.86) | 1.51 (0.09) | 1.65 (0.13) | 2.88 (10^-5.14) | 1.18 (0.39) | 1.08 (0.64) | 4.83 (<10^-16) | 1.22 (0.13) | 0 (0.97) | 1.97 (0.08) |
| Obesity | 1.66 (<10^-16) | 1.33 (<10^-16) | 1.4 (<10^-16) | 1.99 (<10^-16) | 1.66 (<10^-16) | 1.16 (<10^-16) | 1.2 (<10^-16) | 1.58 (<10^-16) | 1.08 (<10^-16) | 1.07 (<10^-16) | 1.25 (<10^-16) | 1.12 (<10^-16) | 1.47 (0.01) | 3.53 (<10^-16) |
| Parkinson’s disease | 0.98 (0.56) | 1.14 (10^-3.93) | 1.18 (10^-10.07) | 1.14 (0.04) | 0.98 (0.48) | 0.92 (0.16) | 0.85 (0.06) | 1.74 (<10^-16) | 0.99 (0.82) | 1.02 (0.47) | 1.27 (10^-8.89) | 1.03 (0.21) | 1.78 (0.21) | 1.09 (0.46) |
| Primary biliary cirrhosis | 2.24 (10^-11.81) | 1.49 (10^-3.9) | 1.59 (10^-7.04) | 2.77 (10^-11.64) | 2.15 (10^-13.41) | 1.77 (10^-4.4) | 2.03 (10^-3.28) | 6.07 (<10^-16) | 1.34 (0.01) | 1.38 (10^-2.83) | 1.36 (0.07) | 1.49 (10^-5.23) | 0 (0.97) | 1.39 (0.2) |
| Sudden cardiac arrest | 0.56 (10^-10.78) | 0.37 (<10^-16) | 0.66 (<10^-16) | 1 (0.99) | 0.63 (10^-10.59) | 0.84 (0.12) | 0.43 (10^-3.83) | 1.58 (10^-6.03) | 0.39 (<10^-16) | 0.34 (<10^-16) | 0.71 (10^-3.71) | 0.34 (<10^-16) | 1.35 (0.77) | 1.22 (0.28) |
| Ulcerative colitis (IBD) | 1.89 (<10^-16) | 1.8 (<10^-16) | 1.72 (<10^-16) | 2.21 (<10^-16) | 1.89 (<10^-16) | 1.77 (<10^-16) | 1.72 (<10^-16) | 2.74 (<10^-16) | 1.85 (<10^-16) | 1.61 (<10^-16) | 1.67 (<10^-16) | 1.66 (<10^-16) | 2.72 (0.03) | 2.35 (<10^-16) |
| Venous thromboembolism | 1.38 (<10^-16) | 1.14 (10^-12.44) | 1.45 (<10^-16) | 1.52 (<10^-16) | 1.26 (<10^-16) | 1.43 (<10^-16) | 1.13 (10^-2.37) | 3.68 (<10^-16) | 1.26 (<10^-16) | 1.09 (10^-6.42) | 1.67 (<10^-16) | 1.16 (<10^-16) | 3.2 (10^-6.3) | 1.67 (<10^-16) |

**Supplemental Table 3. The most common comorbidity prevalence**

|  | Celiac disease | Crohns disease | Ulcerative colitis |
| --- | --- | --- | --- |
| Skin disease cohort | 0.51% | 0.64% | 0.94% |
| Control cohort | 0.22% | 0.36% | 0.52% |
